# Medical and Physical Intelligence

**Published:** 1824-02

**Authors:** 


### MEDICAL AND PHYSICAL INTELLIGENCE.

### ANATOMY AND PHYSIOLOGY.

1. *On a Muscular Bag communicating with the Windpipe, in the Cassowary*, by Dr. Knox.—The Indian cassowary has long been remarkable for the continuation of the cartilaginous rings of the bronchia into the lungs themselves, and for the existence of muscular fibres, after these have ceased. Both are also common to the New-Holland cassowary, whose trachea is much larger and longer than that of the Indian. In the former, likewise, at the fifty-second ring, counting from the glottis, there is found a large muscular bag, about the size of a man's head, into which the windpipe opens by a large orifice, occasioned by a deficiency of a part of the circumference, in about thirteen tracheal rings; or, rather, the rings, instead of closing around to form the tube of the trachea, expand outwards, and are attached to the sides of the bag. This most remarkable, and, so far as I know, unique structure, attracted a good deal of my attention. It has no communication with any of the air-cells. I was at first at a loss to conjecture the use of this bag, and its importance to the animal; but, reflecting on the nature of the country in which the emeu is found, it seemed to me extremely probable that nature, ever watchful of all her works, may have superadded this muscular appendage to the trachea of the New-Holland cassowary, to preserve it amidst those dangers from sudden floods, to which New Holland is particularly exposed. The sandy plains of this extraordinary country are, during a great part of the year, inundated, and become then boundless marshes; and the plains generally are exposed to sudden inundations. The rivers, moreover, running westward from the great chain of mountains, terminate in vast muddy plains or inland marshes. The emeu, forced to seek his food amidst these fens, may, when obliged to have recourse to swimming, (which must very often be the case,) fill the muscular bag of the trachea with air, and thus convert it into a swimming bladder. It may also assist the bird in escaping from his pursuers: but on this I mean not to insist, as the organ is wanting in the Galeated cassowary and in the ostrich, both remarkable for speed of foot. A moment's reflection must convince every one that the bag can only be filled by the expiration of the bird, and that it cannot be dilated by inspiration; or, at least, it is excessively difficult to imagine how inspiration could be prolonged to such an extent as to fill the air-cells of the body, the lungs, and muscular appendage of the trachea. On the other hand, the bird has only to employ the mechanism by which he forces the air into the air-cells and the osseous cavities,—i. e. by closing the glottis and compressing the chest. On doing this, and calling into action the abdominal muscles, the air must of necessity be forcibly driven into the bag of the trachea ; and may, by retaining the glottis shut, be alternately circulated between the lungs, air-cells, and bag of the trachea, giving the bird an additional advantage in running. It may not be altogether uninteresting to add, that the contents of the stomach showed that these birds had been chiefly fed on animal fat.

"This bag is situated in the neck, immediately above the bone called *merry-thought;* it was seen by me in the female, though it is probable that the male also possesses it. It is quite peculiar to the bird, no such appendage having been ever seen attached to the trachea of any of the feathered creation; nor do I know of any thing analogous to it in any other animal, excepting in the cameleon, to the upper portion of whose trachea there is appended a comparatively large membranous bag.

"I stated in my former observations, that I believed it to perform the very important function of enabling the bird to swim, and so preserve life amidst the extensive marshes composing central New Holland, and to escape also from those sudden inundations to which Australasia is generally exposed. It is to be hoped that the observations of naturalists will soon decide on the correctness of this theory. In the mean time, we may remark, that the muscular bag of the trachea may be extremely useful to the bird in running, altogether independent of the function I have assigned to it; for it is evident that, by its means, and the precaution of shutting the glottis, the bird will be enabled to cause the air of one inspiration to pass and repass the lungs, without being necessitated to allow it to escape, in order again to perform the act of inspiration.

"It is now, I think, made evident that the appendage of the trachea in the cassowary of New Holland has not the most distant resemblance to those found in other birds; and that, in thus differing so singularly and mysteriously from the analogous structure of birds of the Old and New Continent, it fully confirms the opinions of some naturalists, that the living productions of Australasia will, when properly examined, be found to present peculiarities altogether wonderful, and perhaps yet, for a long period, quite inexplicable.—(*Edinburgh Philos. Journal.*)

### PATHOLOGY.

2. *Degeneration of Muscles from long Inaction.*—M. Guersent lately presented, to the Royal Academy of Medicine of Paris, an example of that degeneration which has been called *fatty* of the muscles of the breech. A child was affected for three years with a complete contraction of the right lower extremity, the leg being permanently bent on the thigh, and the thigh on the pelvis. He died of the croup. The spinal marrow was healthy, as well as the nerves arising from it. The great gluteus muscle, deprived of its natural red colour, presented the appearance of yellow wax; but the form and direction of the fibre were easily recognised, and the tissue of which these were composed could not be confounded with that of the adipose substance between them. The muscle, then, was not converted into fat, but only the fibrin constituting the basis of the muscular tissue was entirely deprived of colouring matter.—This case is in unison with the observations of M. Beclard, who thinks that this kind of transformation in muscles consists solely in their being deprived of colour and becoming extenuated, while fat accumulates in the interstices between the fibres. In the same individual, the gastronemius muscle of the contracted side was remarkable for its great development, although it was extremely pale.—(*Revue Medicale,* Novembre.)

### THERAPEUTICS AND MATERIA MEDICA.

3. *Illustration of the Utility of the Sulphate of Quinina in Intermittent Fevers.*—De Rossi and Tonelli, two Italian physicians, have made numerous experiments with this substance; the following summary of which is given in the last Number of the Edinburgh Medical and Surgical Journal:

"De Rossi used the sulphate of kina in sixty-four cases; namely, in eight simple tertians, twenty-nine double tertians, two simple quartans, seventeen subcontinued, and eight pernicious intermittents. All of these were cured: fifty had no subsequent paroxysm; seven had only one very slight fit; five, two similar fits; one, several fits; and one passed into a continued remittent fever. One person, affected with quartan for twelve months, recovered after a single slight return of the paroxysm. The quantity of the salt used varied between twelve and seventy-two grains; and, in forty-five cases, did not exceed twenty-four. The remedy was given in pills, and always in the apyretic intervals, in doses of four grains every hour, or every alternate hour; and, in general, to confirm the cure, four grains were given daily for ten days after the last paroxysm. The principal advantages to be derived from it, in preference of the bark itself, are the following:—It may easily be given to children, and to those who have a disgust at bark, or are affected by it idiosyncratically; and it has a rapidity of effect, especially in pernicious and sub-continued intermittents, far surpassing that of the bark. He further proves that large doses produce no bad consequences,—in other words, that it can never act poisonously; that it may be given safely to persons of all ages and temperaments, to puerperal, pregnant, and menstruating females; and that, when it fails, the failure is owing either to the existence of some other morbid cause, or to the sulphuric acid being present in excess, or to the salt being decomposed by pharmaceutic substances intermingled, or by other matters which it encounters in the alimentary canal.

"The observations of Tonelli are equally pointed. He used it in sixty-five cases; namely, in four quotidians, twenty-two simple tertians, thirty-one double tertians, three simple quartans, two double quartans, two subcontinued, and one pernicious intermittent. All of them recovered, except the last, who would not take the remedy till he was insensible and near the point of death. Forty-two recovered without any return of the fit; of the sixty-five cases, only forty-three required a repetition of the first dose, which varied from twelve to eighteen grains. These results, especially those of De Rossi, who used the salt in greater quantity than Tonelli, and in a greater number of bad intractable cases, place the superiority of the sulphate of kina beyond all doubt, both as to convenience and efficacy."—(*Giornale Arcadico di Scienze, &c.* Novembre 1822.)

4. *Inconveniences resulting from the Sulphate of Quinina.*—In an account of the Transactions of the Royal Medical Society at Bordeaux, we find the following remarks upon this medicine.

M. Guitard has treated a *rheumatic* intermittent fever, accompanied with irritation of the stomach: these complaints, exasperated by the use of the sulphate of quinina, disappeared on exhibiting the powder of bark united with magnesia.

M. Guerin, jun. reports the case of a female, seventy-six years of age, who had been poisoned two years previously with verdigris, and whose stomach retained a great portion of irritability: she suffered, last April, from an attack of a quotidian fever, which was exasperated by the employment of the sulphate of quinina, but which yielded to several copious local bleedings upon the epigastric region. The sulphate of quinina is not, therefore, always so innocent as is pretended. M. de St. Crie has remarked, that, when it is prescribed in too large doses in intermittent fevers, it causes a great degree of irritation at the stomach, destructive of the febrifuge power of the remedy.—(*Journal Complémentaire,* Decembre.)

### SURGERY.

5. *Retention of Urine.*—M. Dupasque has published two cases of retention of urine, the leading facts of which are as follow.—The first patient, a valet-de-chambre to a man of opulent family, living well, and not subject to much fatigue or hard work, at the age of forty-five began to be troubled with hemorrhoidal tumors; at fifty-five, these attacks of piles were accompanied with frequent desire to make water, and a difficulty in emptying the bladder, sometimes amounting to complete retention. The catheter was, on this account, used every three or six months; and, though it was introduced easily, a great deal of blood always followed its introduction. The patient had often applied leeches to the part, observing that, by so doing, the attacks of the piles were less frequent, and consequently the retention of urine. In August, 1817, M. Duparcque first saw the patient, who had not had an attack for five months until then: the bladder had then become evident above the hypogastrium, and the patient, with the most violent efforts, could only bring away a few drops of blood and a coagulum or two. The anus was surrounded by hemorrhoids, and a sense of tension at the perineum was complained of. Delirium, nausea, vomiting, and a frequent and hard pulse, were present. The practice employed by M. Duparcque was, to endeavour to pass a catheter, to press upon the region of the bladder, and to draw the stilet in and out several times: nothing but clots of blood, however, were passed. This first trial was with the silver catheter. A gum elastic one was then passed, to which a syringe was adapted; a little warm water was then injected, to wash away the blood supposed to obstruct the escape of the urine. The piston being then withdrawn, a mixture of clots of blood, urine, and water, was discharged. This operation, repeated twice, enabled the operator to empty the bladder, merely by pressing above the pubis. The catheter was left in the bladder, and fifteen leeches applied to the perineum: nevertheless, another collection of clotted blood took place. The syringe was again employed, and twenty more leeches applied. Afterwards no fresh clot of blood appeared; and, by the evening, the urine re-acquired its usual appearance and colour. It should be observed, that cloths wet with *cold oxycrat* had been applied to the thighs and pubes. A similar, but slight, attack took place five months after this; but the leeches and cold applications removed the symptoms in less than twenty-four hours. The patient is now able to avert the retention of urine, by the application of leeches previous to the attack.

The second case is of a similar nature: the patient, however, was only forty-two years of age, and had occasionally been subject to retention of urine for upwards of six years. There was no impediment in the canal, and the flow of blood was but trifling. The presence of hemorrhoidal tumors appeared also in this case to bring on the retention of urine; and the application of leeches and cold bathing to the parts produced a speedy remission of the symptoms. However, after a lapse of six months, in consequence of great irregularities in diet, a violent attack of retention came on, so that only a few drops of blood and some fragments of coagula could be passed: the syringe and injection of warm water was had recourse to, with the same happy effect as in the first instance. Here also an active general treatment was resorted to, and the patient recovered.

The author, in conclusion, after making some reflections on these cases, which we do not deem it necessary to transcribe, attributes the rapid suspension of the hemorrhage to the use of the extract of ratany root, which he administered to the patient in the proportion of one drachm dissolved in four ounces of liquid; after the fifth spoonful of which, he says, the urine was less coloured, and the clots of blood less in quantity.—(*Journal de Medicine*, Novembre.)

6. *On the Use of Injections of Tepid Water into the Bladder in Catarrhus Vesicæ.*—M. JULES CLOQUET lately treated, at the Hospital of St. Louis, a case of very severe catarrhus vesicæ, by means of "irrigations" of tepid water introduced into the bladder. The disease had previously resisted various methods of treatment, and remained perfectly cured at the time this account is dated, eighteen months after his discharge from the hospital. M. Cloquet mentions, however, that he has not always obtained such satisfactory results, many experiencing only an inconsiderable relief from this method of treatment, but that in no case was the disease aggravated.—(*Revue Medicale*, Novembre.)

### CHEMISTRY AND PHARMACY.

7. *Action of Hydrogen upon Platinum.*—Some of the most singular phenomena, lately discovered in this science, are those presented by various preparations of platinum,—as the suboxide, the oxide of the sulphuret, and the metallic powder. When the powder of platinum is subjected to the action of a stream of hydrogen, it becomes first red, and then white hot, and continues in this state as long as there is any hydrogen acting upon it. We have frequently seen this experiment performed, and have examined an extremely ingenious application made of it by Mr. GARDEN, of Oxford-street. By a very simple contrivance, a glass vessel is kept constantly supplied with hydrogen, under a certain degree of pressure, so that it rushes out on turning a small stopcock. Beneath this is placed a little dish, containing the platinum powder, which soon gives out so much heat as to ignite the hydrogen gas. The whole apparatus is so simple, as scarcely to admit of being deranged, and contained within so small a space as to form the most convenient method we have seen of obtaining an instantaneous light. In performing this experiment, it is necessary that the hydrogen be allowed to pass through from one to two inches of atmospheric air, before it arrives at the platinum.

8. *Substitute for Prussic Acid.*—There are few medicines concerning which more contradictory accounts have been published, than those respecting the prussic acid. This is, no doubt, to be attributed in part to the extreme volatility of its nature, and the facility with which it undergoes decomposition, by which means it is difficult to procure it of uniform strength, or to ascertain that it is exhibited in a satisfactory condition. With a view of obviating this evil, and procuring a preparation of a less evanescent kind, MM. ROBIQUET and VILLERMÉ have performed a variety of experiments; the results of which have led them to propose the hydro-cyanate of potass, or the cyanuret of potassium, as a substitute in practice for the simple hydrocyanic acid. The fact of its less easy decomposition, on dilution with water and exposure to light, seems to have been satisfactorily shown; but the question whether the action of this powerful medicine upon the animal frame, when combined with an alkali, be identical with that of the pure acid, or so similar as to render it admissible as a substitute, remains to be ascertained by future observation. That this substance exerts a powerful and deleterious influence, producing death in a manner analogous to the prussic acid itself, was ascertained by the sacrifice of a number of dogs, guinea-pigs, birds, &c. which were speedily killed by it; and the general conclusions of MM. Robiquet and Villermé are as follows:

"1st. That the effects of the pure hydro-cyanate of potass, as manifested in our experiments, are similar to those of the hydro-cyanic or prussic acid.

"2d. That the employment of the hydro-cyanate of potass, prepared extemporaneously by the solution of the cyanuret of potassium, appears capable of being substituted with advantage for the hydrocyanic acid, such as has generally been used up to the present time; and that this deserves attention.

"3. That it would be necessary to ascertain whether the new medicine which we propose may not produce, upon the animal economy, other effects than the prussic acid.

"4. And that, in case of its producing others, these would require to be accurately inquired into, to ascertain whether they be hurtful or otherwise."—*(Journal de Physiologie.)*

---

### MISCELLANEOUS.

9. Mr. EARLE *on Injuries near the Shoulder-Joint.*—In our last Number, (at page 50,) in which we gave some account of an apparatus, the invention of Mr. Earle, adapted to prevent the motion of the arm and shoulder in injuries of the parts connected with that joint, an error of so much importance has occurred, in consequence of the omission of a short paragraph, that we are induced, both in justice to Mr. Earle, as well as for the sake of our readers, to correct it in the most clear and explicit manner. The sentence omitted in our last Number is the following:

With a view to obviate the difficulties attending the modes of procuring the perfect rest of the limb, heretofore in use, Mr. Earle has constructed the following apparatus, which he has *successfully employed* in fractures of the clavicle, and fractures of the neck of the scapula.—The concluding sentence, then, only alluded to dislocations of either extremity of the clavicle; cases which are so rarely perfectly restored, that Sir Astley Cooper is in the habit of stating, at the conclusion of his lecture on this subject, that, after the utmost care in the treatment, some slight projection and deformity will remain; and therefore inculcates the propriety of stating this to the patient in the first instance. This, therefore, is the case which Mr. Earle has not yet had an opportunity of employing his bandage on, but from which he hopes for a more perfect restoration of the limb, as the deformity complained of can only depend on the imperfect mode in which the upper extremity has been hitherto secured during the cure.

10. *Effects produced on the Crew of a Man-of-War by the Bursting of Vessels containing Quicksilver.*—A very curious and interesting account is given by Dr. BURNETT, one of the medical commissioners of the navy, of the effects produced on-board a King's ship by the bursting of various vessels containing quicksilver, by which that substance became dispersed all over the vessel. In the space of three weeks, two hundred persons were affected with salivation, ulcerations of the mouth, accompanied, in many instances, with partial paralysis and bowel-complaints. Almost all the live stock on-board were likewise affected, consisting of sheep, pigs, goats, poultry, mice, cats, a dog, and a canary-bird. The vapour was very deleterious to those who had any tendency to pulmonic affections. Only two died from the ptyalism, gangrene having taken place in their cheek and tongue: they had previously lost all their teeth. In the case of a woman who was confined to bed in the cock-pit with a fractured limb, not only were all the teeth lost, but many exfoliations also took place from the upper and lower jaws. The mercury showed effects upon the ship herself, by the decks being covered with a black powder, in which, however, quicksilver was never discovered in a globular state. Several tons of this metal had escaped from its packages, and became diffused through the ship, by the constant motion of which it had become oxydised, and probably impregnated the atmosphere of the vessel with mercurial vapours. It is of importance to know that sulphur, given internally in large quantity, had no effect in relieving the symptoms, but served to aggravate the derangement of the bowels, and to bring on tenesmus: its external application was especially unavailing. The only plan which produced effectual relief was removal from the ship, with the frequent use of small doses of neutral salts and detergent gargles.

"Various opinions were entertained of the manner in which the systems of the sufferers were brought under the influence of the mercury. By some, it was supposed to have originated from the use of the bread and other provisions, with which the mercury had mixed itself; and to such an extent was this opinion carried, that I find, by reference to official documents in the Victualling Office, 7,940 pounds of biscuit were condemned as unserviceable from having *quicksilver mixed with it.*

"By others, amongst whom was Mr. Plowman, the surgeon, it was considered to have arisen from inhaling the mercurialised atmosphere; and, from the preceding details, I think there cannot remain a doubt that this opinion was the true one.

"It is well known that mercury, in its native state, has often been administered in very large doses, in cases of obstinate constipation, without producing any specific effect on the system; merely removing the affection by its specific gravity. I have, however, reason to believe, from the accounts of Orfila and others, that if the mercury was to be retained in the intestines for some time, and thus subjected to the action of the contents of the stomach and bowels, a part might become oxydated, and, being conveyed into the system by means of the absorbents, would there show its specific effects.

"But, after the removal of the provisions, &c. at Gibraltar, many fresh cases occurred, and many relapses, amongst those who had been cured out of the ship, took place on their return to duty on-board, which effectually destroys the probability of this having been the cause of the succeeding ptyalism, and other morbid affections.

"It only remains for me to offer my opinion of the manner in which the system became saturated by the mercury, and this I conceive to have been effected by inhaling the mercurial vapours; the quicksilver, being then in the most perfect state of division, was readily taken up by the absorbents of the lungs, and soon showed its influence on the system generally. This idea is very much strengthened by the effect which was produced on the animals on-board, already mentioned, as well as by the circumstance of a great number of men being attacked after the ship was cleared at Gibraltar, and till she arrived in a more northern latitude."—*Transact. of Royal Society*, 1823, Part ii.)

11. *Gymnastics.*—A little work by Mr. CLIAS, who has established his system of gymnastic instruction at the Royal Military Asylum, under the auspices of H. R. H. the Duke of York, has been put into our hands: we can now do little more than point out the general merits of this gentleman's plan. Captain Clias has long been known on the continent as the active promoter of all those exercises tending to develop and increase the physical powers of man; and we find prefixed to his work a very handsome testimonial of the benefits derived from his instructions at the Orphan School at Berne, where our author taught his system for five years. This testimony is in the form of a letter from the president of that establishment (Mr. Stettler), who observes, that these exercises have produced the happiest effects on the health, and consequently on the *morals*, of the children. This last consideration is, in our opinion, so important, and so true, that we shall take an early opportunity of laying a more detailed account of Captain Clias's plan before our readers; for, although we believe that, in this country, bodily exercises are much more in use, especially in our scholastic establishments in the country, than our author seems to be aware of, still these are left too much to the discretion of the pupils themselves, and the powers of the body are consequently not called forth in the regular and systematic method taught by our author, and which is not only well calculated to give the body its full degree of strength and activity, but, by the mode of teaching, precludes almost the possibility of the occurrence of any accident.

---

OBITUARY.—Lately deceased, EDWARD GRAINGER, Esq. Lecturer on Anatomy, &c.—We regret that the Communication made to us on this subject, was too late for us to avail ourselves of it.

---

### MONTHLY CATALOGUE OF MEDICAL BOOKS.

### METEOROLOGICAL JOURNAL,

*From December* 20, 1823, *to January* 19, 1824.

By Messrs. William Harris and Co. 50, Holborn, London.

| | MOON. | Rain gauge | THERM. | | | BAROM. | | DE LUC'S HYG. | | WIND. | | ATMOSPHERIC VARIATION. | | |
|---|---|---|---|---|---|---|---|---|---|---|---|---|---|---|
| | | | 9 A.M. | MAX. | MIN. | 9 A.M. | 10P.M. | 9 A.M. | 10P.M. | 9 A.M. | 10P.M. | 9 A.M. | 2 P.M. | 10P.M. |
| Dec | | | | | | | | | | | | | | |
| 20 | | .60 | 38 | 42 | 35 | 29.20 | 29.10 | 89 | 90 | SE | SE | Rain | Fair | Cloud. |
| 21 | | | 38 | 40 | 36 | 29.12 | 29.24 | 87 | 90 | S | WSW | Fair | Fine | Rain |
| 22 | | | 38 | 40 | 35 | 29.40 | 29.70 | 85 | 86 | W | W | Overc. | Fair | Fair |
| 23 | | | 39 | 40 | 37 | 29.64 | 29.82 | 85 | 90 | W | W | Rain | Rain | Rain |
| 24 | ☾ | | 38 | 46 | 40 | 29.82 | 29.88 | 90 | 92 | W | WSW | | | |
| 25 | | | 46 | 49 | 43 | 29.84 | 29.70 | 90 | 90 | SW | S | Rain | | |
| 26 | | .55 | 44 | 48 | 44 | 29.68 | 29.47 | 90 | 90 | S | S | Cloud. | Fair | Fair |
| 27 | | | 45 | 46 | 39 | 29.14 | 29.14 | 89 | 85 | WSW | SW | Fair | Fine | |
| 28 | | | 43 | 49 | 40 | 29.34 | 29.10 | 80 | 84 | SW | SW | Fair | Rain | Rain |
| 29 | | | 42 | 45 | 42 | 29.10 | 29.00 | 70 | 74 | SSW | S | | Fine | |
| 30 | | | 43 | 44 | 40 | 29.00 | 39.12 | 70 | 75 | SSW | SSW | Fine | | Fair |
| 31 | | | 42 | 45 | 42 | 29.40 | 29.59 | 78 | 78 | WSW | WSW | | | |
| Jan. | | | | | | | | | | | | | | |
| 1 | ● | | 43 | 45 | 43 | 29.20 | 29.55 | 79 | 75 | SSW | SSW | Overc. | | |
| 2 | | | 44 | 47 | 40 | 29.25 | 29.60 | 69 | 70 | WSW | WNW | | Fair | Cloud. |
| 3 | | | 40 | 43 | 33 | 30.05 | 30.24 | 78 | 80 | WNW | NW | Fine | Fine | Fine |
| 4 | | | 34 | 35 | 33 | 30.35 | 30.35 | 89 | 89 | NNW | NW | Foggy | Foggy | Foggy |
| 5 | | | 35 | 37 | 34 | 30.36 | 30.27 | 85 | 85 | NNW | SW | | | |
| 6 | | | 35 | 40 | 34 | 30.12 | 30.08 | 75 | 75 | SW | SW | Fair | | Fair |
| 7 | | | 35 | 37 | 34 | 30.20 | 30.22 | 72 | 75 | NNW | N | Fine | Fine | Fine |
| 8 | | | 33 | 38 | 35 | 30.20 | 30.12 | 90 | 87 | NNW | WSW | Foggy | | |
| 9 | ☽ | | 34 | 43 | 42 | 30.10 | 30.06 | 85 | 90 | SW | SW | Overc. | Overc. | Overc. |
| 10 | | | 42 | 44 | 39 | 30.00 | 29.43 | 90 | 92 | SW | SW | Misty | Misty | Rain |
| 11 | | .65 | 41 | 39 | 33 | 30.13 | 30.30 | 89 | 85 | N | N | Fine | Fine | Fine |
| 12 | | | 33 | 36 | 28 | 30.33 | 30.33 | 90 | 92 | NNW | NW | Foggy | | Fair |
| 13 | | | 30 | 32 | 25 | 30.34 | 30.31 | 90 | 88 | NW | NW | | Foggy | Foggy |
| 14 | | | 28 | 32 | 29 | 30.25 | 30.26 | 93 | 90 | NW | NW | | | |
| 15 | | | 35 | 36 | 32 | 29.35 | 30.24 | 87 | 85 | NW | N | Overc. | Snow | Cloud. |
| 16 | ○ | | 34 | 36 | 30 | 30.41 | 30.43 | 77 | 80 | NW | NW | Fine | Fair | Fair |
| 17 | | | 30 | 37 | 30 | 30.40 | 30.32 | 78 | 80 | W | W | Fair | Fine | Foggy |
| 18 | | | 37 | 39 | 35 | 30.28 | 30.10 | 85 | 87 | W | NW | | | |
| 19 | | | 40 | 40 | 38 | 30.10 | 30.02 | 90 | 93 | W | W | Rain | Cloud. | |

The quantity of rain fallen in the month of December, was 1 inch and 15.100ths.

